# Prevalence of Anxiety and Depression Symptoms among University Teachers in Ethiopia during the COVID-19 Pandemic

**DOI:** 10.3390/healthcare12161649

**Published:** 2024-08-19

**Authors:** Tefera Tadesse, Martin R. Fischer, Getu Ataro, Shewatatek Gedamu, Marema Jebessa, Almaz Mamaru, Matthias Siebeck

**Affiliations:** 1 Institute of Educational Research (IER), Addis Ababa University, Addis Ababa P.O. Box 1176, Ethiopia; 2 Institute of Medical Education, LMU University Hospital, LMU Munich, Pettenkoferstr. 8A, 80336 Munich, Germany; martin.fischer@med.uni-muenchen.de (M.R.F.); matthias.siebeck@med.uni-muenchen.de (M.S.); 3 Department of Anaesthesia, College of Medicine and Health Sciences, Hawassa University, Hawassa P.O. Box 05, Ethiopia; getuataro@hu.edu.et; 4 Institute of Health, Jimma University, Jimma P.O. Box 307, Ethiopia; gedamuwonde@gmail.com; 5 School of Medicine, College of Health Sciences, Addis Ababa University, Addis Ababa P.O. Box 9086, Ethiopia; marema.jabessa@aau.edu.et; 6 Department of Psychiatry, College of Health and Medical Sciences, Bahir Dar University, Bahir Dar P.O. Box 79, Ethiopia; aem.alma@gmail.com

**Keywords:** anxiety, depression, university teacher, Ethiopia, COVID-19

## Abstract

A growing body of evidence suggests an increased prevalence of anxiety and depression among teachers during the COVID-19 pandemic. However, there is little evidence in research documenting the extent of anxiety and depression in the university teacher population and how these relate to feelings of loneliness. This study aims to explore the prevalence of anxiety and depression symptoms among university teachers, identify differences, and further examine the relationships between loneliness and symptoms of anxiety and depression. The study participants included university teachers in the College of Health and Medical Sciences from four purposefully selected public universities who completed a survey questionnaire consisting of items that measured anxiety and depressive symptoms as well as loneliness. The final sample included 148 participants (45 participants [30.4%] located in the center or capital; 125 (83.1%) men; and 90 [62.5%] taught both online and face-to-face). The university teachers’ sample mean age = 39.07 had an SD = 7.67. As per the findings of this study, the prevalence of anxiety and depression symptoms was significant (11% and 12.3%) among university teachers in Ethiopia during the COVID-19 pandemic. Also, this study found a significant association between these symptoms and loneliness. Therefore, incorporating relevant strategies to promote mental well-being and targeting individuals who felt lonely were essential for overcoming health-related burdens. Universities should equip teachers with resources to prevent mental health issues and offer need-based counseling services to alleviate them.

## 1. Introduction

### 1.1. Background

Anxiety and depression are important at a symptomatic level [1]. Scholars have defined anxiety in various ways, but generally, it denotes an affective, physiological, cognitive, and behavioral state [2]. Anxiety may happen from the perception of a threat [3]. Contrary to this, depression has been defined as a multifaceted state [4] that originates from a perception of an important loss or threat of such a loss [1]. Like anxiety, depression has emotional, cognitive, behavioral, and physiological components. Unlike anxiety, depression entails the components of avoidance, withdrawal, and diminished activity [5]. Depression symptoms cause saddened feelings and/or a loss of interest in activities a person once enjoyed. It can lead to different emotional and physical problems and can decrease a person’s ability to function at work and at home.

Across all psychiatric disorders, comorbidity, which represents the simultaneous presence of two or more medical conditions in a person, is common or usual [4]. This comorbidity applies to anxiety and depressive disorders and their symptoms [5]. Hence, anxiety and depression are highly comorbid [6], and their disorders and symptoms are frequently inseparable [7]. After reviewing the existing data, the researchers concluded there was an overlap between perceptions of depression and anxiety [6].

Loneliness is a psychological state associated with deficiencies in a person’s social relationships [8]. While loneliness is not a facet of mental health by itself, research indicates that loneliness is a key predictor of mental health, including anxiety and depression [9]. Indeed, a growing body of evidence indicates that the current pandemic crisis intensifies loneliness as a risk factor for anxiety and depression [10,11].

The COVID-19 pandemic has resulted in one of the most outrageous health and economic crises of the 21st century, and its multidimensional influence surpasses the personal, institutional, and national levels. University teachers’ academic and research work at universities has been seriously affected [12,13]. University teaching in postgraduate programs is demanding, particularly during the pandemic season, as teachers face further challenges due to the demands of the new normal and new ways of delivering instruction, including online and blended learning, while they also have limited access to social support [14].

Recent studies have pointed out that during the COVID-19 pandemic, university teachers have suffered a lot in adapting or transforming instructional delivery to online learning and blended learning modalities while maintaining the demands of the new normal [15]. This demanding change and the resulting crises have often been accompanied by symptoms of anxiety and depression [16,17,18,19,20,21].

### 1.2. Statement of the Problem

University academia is an intrinsically motivating job, but it may be the source of negative affective conditions, leading to mental health issues. Before the COVID-19 pandemic, mental health issues, particularly for adults between the ages of 25 and 49, were the leading causes of the global health-related burden, and depressive and anxiety disorders were leading contributors to this burden [22]. COVID-19 placed serious emphasis on the professional life and mental well-being of university teachers and students, exacerbating the factors of poor mental health among university teachers [13,15]. Both anxiety and depression symptoms may have consequences for the long-term health of university teachers and, as a result, could lead them to take increased sick leave, absenteeism, and poor work performance.

The COVID-19 pandemic’s full impact on university teachers’ mental health has not been well understood yet. The need for up-to-date information on the mental health impacts of COVID-19 in a way that informs health system responses is very much needed to mitigate these challenges [23,24]. However, there is minimal research attention directed to the mental health of university teachers. The minimal studies available have identified that factors such as degree qualification, academic rank, instructional type, and university location critically affect university teachers’ anxiety and depression symptoms [14,15]. Additional factors identified include gender, age, employment stability, educational attainment, and parental status as influences on anxiety and depression symptoms [25,26,27,28].

Loneliness is thought to be particularly related to anxiety and depression symptoms [29], and even recent evidence shows that this relationship has intensified during the COVID-19 pandemic [11,30,31]. The ongoing COVID-19 pandemic has significant implications for mental health around the globe. University teachers and postgraduate students are among the most vulnerable groups to mental health issues due to academic pressure exacerbated by the pandemic-induced health and economic crises [32,33,34]. However, the effects of the pandemic on anxiety and depression symptoms, particularly among university teachers in Ethiopia, are unknown. The purpose of this study was to explore the prevalence of anxiety and depression symptoms, identify differences, and examine relationships with loneliness in Ethiopian universities during the COVID-19 pandemic. This study answered the following three research questions:

(1) What is the prevalence of anxiety and depression symptoms in university teachers in Ethiopia during the COVID-19 pandemic?
(2) Are there differences in the symptoms of anxiety and depression that university teacher participants report based on gender, age, academic rank, instructional type, university location, and state of loneliness?
(3) Does loneliness predict symptoms of anxiety and depression that teachers report in Ethiopian universities during the COVID-19 pandemic?

### 1.3. Hypotheses

Based on the previous literature and an emerging body of evidence on teachers’ mental health during COVID-19 [24,35,36], the authors hypothesized that university teachers’ anxiety and depressive symptoms during the COVID-19 pandemic may differ by gender, age, teaching experience, academic rank, instructional type, university location, and loneliness.

Feeling lonely and having mental health problems, such as anxiety and depression, are strongly linked [37]. Feeling lonely correlates with anxiety since the individual mind cannot relax, which then transfers to the body [38]. Also, loneliness is associated with depressive symptoms. Research shows that loneliness predicts increases in depressive symptoms [39]. The research findings sought positive correlations between mental health and feelings of loneliness before and during the COVID-19 pandemic [40]. Based on research evidence and the links between loneliness, anxiety and depression, the premise of our research contemplates loneliness as a factor that may influence a university teacher’s mental health, including symptoms of anxiety and depression.

## 2. Materials and Methods

### 2.1. Study Design

In this study, the authors used a cross-sectional survey design, collecting quantitative data from university teachers in four purposefully selected public universities in Ethiopia. This design was found to be suitable for the study focus, investigating the prevalence of mental health among university teachers and examining predictors during the COVID-19 pandemic.

### 2.2. Study Participants and Sampling

The target population included academic members from the four selected universities who offered courses or advised postgraduate students in the major fields of health and medical sciences during the 2021–2022 academic year. A three-stage sampling procedure was used to select the study participants. In the first stage, four public universities were purposefully selected based on their generation of establishments and geographic location representation in the country. In the second stage, the health and medical sciences college was purposefully selected because the pandemic had a greater impact on educators in this college compared to other academic members of the university. Health and medical sciences educators often work in high-stress environments, even under normal circumstances. During the COVID-19 pandemic, stress likely increased for them due to the nature of their subject matter directly relating to the pandemic. Hence, these educators may have felt additional pressure to stay updated with rapidly changing information and address students’ heightened concerns about the pandemic. In stage three, from each university, the sample included teachers/advisors (*n* = 40), using stratified random sampling methods with the strata, including program type, academic rank, and gender according to the existing numbers in the Human Resource Division.

Statistical methods, using a single proportion formula, determined a sample size of 160 from a study population of 800 to ensure a 95% confidence level, 80% power, and an adjusted margin of error of approximately 0.1 (10%). This method ensured statistical significance while accommodating practical constraints. A sample of 160 respondents completed the survey questionnaire, and 148 usable questionnaires were retained after accounting for missing data.

### 2.3. Measures

Demographic information: In this study, demographic information was collected through the self-reported questionnaire regarding university teachers’ gender and age. Gender (identified as female and male) and age in years were self-reported by the participant teacher in the survey questionnaire.

Contextual factors: also, other contextual information such as academic rank, the type of instruction delivered during the academic year 2021–2022, and university location were collected via the self-reported questionnaire.

Anxiety and depression symptoms: Teachers were screened for anxiety symptoms using the short form Generalized Anxiety Disorder-2 (GAD-2) two-item scale [41]. Additionally, they were screened for depression symptoms using the short-form Patient Health Questionnaire-2 (PHQ-2) two-item scale [42].

In the original versions of these two screening tools, the items represented core anxiety or depression symptoms, with scores ranging from 0 to 6. However, the scale was modified into a five-point scale based on the pilot results and experts’ comments received during the questionnaire piloting.

The PHQ-2 asks about the frequency of depressed feelings over the past two weeks. Similarly, GAD-2 asks about the frequency of anxious feelings over the same period. The scale ranges from 0 (not at all) to 4 (nearly every day) for each question. The sum score threshold was modified to determine the cut-off scores for the likelihood of anxiety or depressive symptoms based on the changed scale. The responses for two questions in each scale were summed. If the score for PHQ-2 was ≥4 (out of 8), we considered the likelihood of depression symptoms. Similarly, if the score for GAD-2 was ≥4 (out of 8), we considered the likelihood of anxiety symptoms.

Following a similar rule for formulating the cut-off score for anxiety and depression symptoms, if the teacher’s response was ≥2 (out of 4), we considered the likelihood of loneliness. In this analysis, anxiety was coded as the event. For numeric categorical predictors, coded as 0 and 1, the reference level was the level with the least numeric value (0).

Loneliness: A single-item direct measure of loneliness was utilized by asking participants, “How often do you feel lonely?” They used a scale ranging from 0 (not at all) to 4 (feeling every day). This specific measure has been employed in various studies to evaluate loneliness [43,44,45,46], with some of them focusing on the development and validation of a short scale for measuring loneliness, including single-item measures.

Prior to the main data collection, the questionnaire items were first reviewed by a measurement and evaluation expert and health professional educator for content validity, and their comments were incorporated to enrich the items. Estimates of internal consistency for the two items, anxiety and depression symptoms, were calculated using a pilot sample of reliability for the teachers in the College of Education and Behavioural Sciences at Addis Ababa University (*n* = 25). Cronbach Alpha was used to determine the internal consistency between each set of items. Estimates of internal consistency for the pilot study exceeded α > 0.70. These alpha coefficients are acceptable for higher education research in the literature [47].

### 2.4. Study Procedures

The study’s data were collected from 1 June 2022 to 14 July 2022, adjusting the data collection processes to ensure safety and compliance with COVID-19 health guidelines. All data collectors used face masks when gathering data in person, and they routinely sanitized and cleaned communal areas and equipment.

Data collection was handled by trained data collectors. Prior to data collection, each teacher participant was asked to provide consent to participate. This was facilitated by giving general information about the study. The participants were allowed to ask questions for clarity, and they were encouraged by the data collectors to provide their genuine responses. The completed questionnaires were collected in the strictest confidence and were not identified with each participant individually. Participation was voluntary, and teacher participants were free to choose not to answer any question or withdraw from the survey at any time.

### 2.5. Data Analysis

In this study, all statistical analyses were completed using SPSS Version 26. Specifically, the collected survey data were analyzed at four levels. First, calculating proportions or percentages was found to be useful, including the proportions of university teachers who had depression or anxiety symptoms in our sample. In calculating proportions and percentages, the number of teacher participant samples was simply taken and divided by the sample size. Then, the chi-square tests were used to identify the proportion of participants with depression and anxiety symptoms across a range of groups categorized by gender, age, academic rank, instructional type, university location, and loneliness. Additionally, binary logistic regression analyses were performed. For a predictive variable, loneliness was entered into a two-binary logistic regression analysis to measure the outcomes of interest, including anxiety and depression symptoms.

## 3. Results

The study results are presented in three sections. The first section presents the descriptive statistics, which illustrate the overall prevalence by frequency and percentage as reported by the teacher participant sample. The second section analyzes anxiety and depression symptoms, highlighting differences across selected characteristics at the personal, instructional, and institutional levels. The third section reveals the outcomes of a binary logistic regression analysis that predicts anxiety and depression symptoms.

The results show three sections. First, we present descriptive statistics, indicating the overall prevalence by frequency and percentage. Second, anxiety and depression symptoms are analyzed to show the differences across their selected characteristics at the personal, instructional, and institutional levels. Third, the results of a binary logistic regression analysis are presented for predicting anxiety and depression symptoms, as reported by the teacher participant sample.

The gender composition includes 17% female and 83% male. The mean age was 39.07 years, with a standard deviation of 7.67. The program type included major health fields, including health sciences (*n* = 47), medical sciences (*n* = 77), and public health sciences (*n* = 24). In terms of academic rank, assistant professors (*n* = 104), associate professors (*n* = 36), and professors (*n* = 7) were included. Also, the teaching experience of the sample ranged from year 1 to 38, with an average of 11.4 years of teaching experience and a standard deviation of 7.6. Moreover, the sample included teachers who taught in universities, with 45 identifying their current university in the capital or center and 103 in the regional university. For the instructional type, the sample included 90 teachers who taught in a blended mode and 54 teachers who taught face-to-face.

In Table 1 and Table 2, it is clear that 11.03% of university teachers screened positive for anxiety symptoms, and 12.33% screened positive for depression symptoms. A reliability analysis of the items for the anxiety and depression symptoms was conducted as a preliminary step. The results for the current sample indicate that the anxiety and depression symptom items had reliability scores of 0.76 and 0.83, respectively (Table 1 and Table 2).

As shown in Table 1, none of these chi-square tests were found to be significant except for the proportion of anxiety symptoms across states of loneliness. Concerning loneliness, a chi-square test of independence showed that there was a significant association between feelings of loneliness and experiencing anxiety symptoms, *X*^2^ (1, *n* = 145) = 44.76, *p* < 0.000, with an effect size of 0.56. A further examination of the proportion differences revealed that the proportion of university teachers without feelings of loneliness had a higher prevalence compared to those who did not have symptoms of anxiety. The evidence presented in this study shows that university teachers without feelings of loneliness had a 7.63 times lower probability of reporting anxiety symptoms compared to those with feelings of loneliness. In summary, those who did not feel lonely were more likely than those with feelings of loneliness to have non-anxiety symptoms.

As shown in Table 2, none of these chi-square tests were found to be significant except for the proportion of depression symptoms across states of loneliness. Concerning loneliness, a chi-square test of independence showed that there was a significant association between feelings of loneliness and depressive symptoms, such as *X*^2^ (1, *n* = 146) = 7.84 and *p* = 0.005, with an effect size of 0.23. A further examination of the proportions revealed that the proportion of university teachers who did not feel lonely had a higher prevalence of not experiencing symptoms of depression. Those university teachers who did not feel lonely had a 7.79 (95% CI 0.73, 1.07) times lower probability of reporting feelings of depression symptoms than those who did not report depression symptoms. Again, those who do not feel lonely had a 7.39 (95% CI 0.69, 1.03) times higher probability of reporting not feeling depression symptoms compared to those who felt lonely. Similarly, university teachers who did not feel lonely had a 7.15 (95% CI 0.63, 0.97) times higher probability of reporting not feeling depression symptoms than reporting feeling depression symptoms. In summary, university teachers who do not feel lonely are more likely than those who feel lonely to experience feelings of non-depressive symptoms.

Two separate binary logistic regressions were conducted to analyze how loneliness predicts anxiety and depression symptoms. The results of this analysis support a general model, which suggests that loneliness predicts the mental health of university teachers. Table 3 shows the outcomes of the binary logistic regression analysis.

As shown in Table 3, a university teacher with feelings of loneliness has approximately 3.46 times higher odds of experiencing anxiety symptoms compared to a university teacher without feelings of loneliness. Feelings of loneliness were significantly and positively associated with higher levels of anxiety symptoms during COVID-19, with an odds ratio of 31.89 (95% CI, 8.41–120.83). Similarly, higher levels of depression symptoms during COVID-19 were significantly and positively associated with feelings of loneliness, with an odds ratio of 5.09 (95% CI, 1.48–17.47).

According to Table 3, it is predicted that university teachers who feel lonely will have symptoms of anxiety that are about 3.46 times more prevalent and symptoms of depression about 1.63 times more prevalent compared to those who do not experience loneliness. Therefore, if two university teachers have different levels of loneliness, the individual who feels lonely is expected to have a chance of experiencing symptoms of anxiety that are 3.46 times higher and symptoms of depression 1.63 times higher than the individual without feelings of loneliness.

## 4. Discussion

This study is the first to explore the prevalence of anxiety and depression among university teachers in Ethiopia during the COVID-19 pandemic. This study identified the prevalence of anxiety and depression symptoms among 148 university teachers using cross-sectional survey data. Additionally, this study investigated differences in the proportion of university teachers reporting symptoms of anxiety and depression across gender, academic rank, instructional type, university location, and feelings of anxiety. Furthermore, this study examined the predictability of loneliness for symptoms of anxiety and depression among university teachers.

A study on the mental health of nursing professors during the COVID-19 pandemic revealed that 9.4% of them experienced moderate-to-severe depression, and 18.7% experienced anxiety [20]. In support of these results, the current study provides empirical evidence, highlighting the substantial impact of the COVID-19 pandemic on the mental health of university teachers in Ethiopia and identifies loneliness as a specific factor that exacerbates these conditions. However, a review of the international academic literature in this area indicates the presence of substantial differences among studies in terms of prevalence as well as associated factors. In some studies, factors such as gender, age, employment permanency, educational attainment, and parental status were identified as influencing anxiety and depression symptoms [17,49]. Also, female academics, particularly those experiencing a decrease in household income and those with pre-existing psychiatric comorbidities, were suggested to be more susceptible according to other studies [25,26].

Although a direct comparison of the reported prevalence of anxiety and depressive symptoms before COVID-19 was not possible due to a lack of empirical evidence as well as methodological issues, an increase in the anxiety and depressive symptoms was reported during the COVID-19 pandemic in Brazil [50], Mexico [51], and Spain [14,52]. Based on a scoping review and meta-analysis, researchers reported that the prevalence of anxiety ranged from 10% to 49.4% [52,53,54,55], and depression ranged from 4% to 77% among teachers during the COVID-19 pandemic [53,55,56]. The prevalence of anxiety (11%) and depression (12.3%) symptoms reported by the teacher participants in the current study correspond to the lower margin of the reported ranges of anxiety and depression for the literature in this field. However, this reported prevalence is consistent with the evidence collected from university professors. For example, the prevalence of anxiety among teachers employed at a university ranged from 10% in a rapid systematic review and meta-analysis [19] to 13% in China and the USA [49,53]. These symptoms may be attributed to the sudden switch to online learning and teacher overload, among other factors.

The literature also reveals that, concerning the prevalence of anxiety by gender, a higher prevalence of anxiety was recorded in females across many countries around the world [13,17]. However, the results of the current study did not support this. Concerning the prevalence of anxiety and symptoms by age, teachers in Spain and the USA with older age had a lower prevalence of anxiety [52,53], and Li, Miao [49] found no difference in the average age between Chinese teachers with anxiety and without anxiety. In a study, the prevalence of depression was similar between age groups in Spain [52]. In a similar vein, another study found that teachers aged over 40 years had a lower prevalence rate for depression [53]. In the study by Ozamiz-Etxebarria, Dosil Santamaría [52] among Spanish teachers aged over 46 years, they found this age group to have a lower prevalence of anxiety. Similar results for university teachers in the USA were presented by Evanoff et al. [53]. Other studies identified cases where demographic variables such as gender and age were significant predictors of anxiety and depression symptoms, suggesting that being female and younger significantly and positively predicts higher anxiety and depression symptoms among teachers in the UK [35]. The findings of the current study did not support this evidence.

The authors believe that teaching experience did not necessarily lead university teachers to have good mental health during the COVID-19 pandemic, as new teaching requirements and experiences, such as virtual teaching, new technology, and adapting lesson plans, impacted experienced teachers equally, as well as those with less experience. This contrasts with previous studies, which found a link between university teachers’ mental health and teaching experience [53].

Regarding the prevalence of anxiety to the degree of academic education, a similar prevalence was found in a previous study in 12.7% of those with bachelor’s degrees and 13.4% of those with master’s degrees in China [49]. Consistent with previous studies, in the current study, there was no significant difference in the prevalence of anxiety and depression symptoms by academic rank (see Table 1 and Table 2).

When the results of this study were examined for university teachers’ differences in anxiety and depression symptoms, loneliness was the only significant factor that was found. Lonely teachers have a higher chance of experiencing anxiety and depression symptoms. Several studies have shown that participants who reported higher levels of loneliness also displayed higher levels of anxiety and depression symptoms [39]. Consistent with previous research, the current study found a significant association between loneliness, anxiety, and depressive symptoms. This study suggests that university teacher participants with higher levels of loneliness may experience moderate-to-minor differences in anxiety and depression symptoms, with loneliness playing a role in causing an intermediate difference in anxiety and a small difference in depression [57]. This corroborates with the findings reported in the literature on this field.

The prediction of loneliness on anxiety and depressive symptoms during the COVID-19 pandemic is disturbing from the perspective of the Evolutionary Theory of Loneliness (ETL) [58], which posits that the prolonged loss of reliable social bonds can result in self-preservation bias and implicit attentiveness toward threats. In the context of university teachers, this may provoke further disconnection from others and, in the longer term, can have a harmful impact on the mental health of university teachers [59]. Therefore, public policies such as physical activity promotion and strategies to reduce the economic strains caused by the COVID-19 pandemic are needed to mitigate the impact of the pandemic on mental health. Technology-based interventions are promising to ease the burden of mental health in university teachers [60].

## 5. Conclusions

This study explored the prevalence of anxiety and depression symptoms and differences based on gender, age, academic rank, teaching experience, instructional type, and university locations in Ethiopia in the context of COVID-19. The findings of this study suggest that university teachers experienced anxiety and depression symptoms during the COVID-19 pandemic. Moreover, the findings suggest that the prevalence of anxiety and depression had a significant association with loneliness. Also, loneliness predicts teachers’ symptoms of anxiety and depression differentially, and the prediction of loneliness is higher for anxiety than for depression symptoms.

The mental health of teachers who teach or advise in postgraduate programs during the COVID-19 pandemic is a serious issue due to its multifaceted impact on education quality, student support, and the future healthcare workforce. Addressing these mental health concerns requires a multifaceted approach, including the availability and effectiveness of support systems and technological resources and recognizing and mitigating mental health predictors to ensure the well-being and productivity of university teachers.

University administrators need to monitor teachers’ mental health and understand the potential factors associated with it. Since loneliness can increase symptoms of anxiety and depression among university teachers, it is believed that developing strategies to mitigate loneliness and increase institutional support can help to overcome mental health problems. University teachers are frontline workers, and universities should monitor teacher anxiety and depression and provide continual support. To overcome health-related burdens, it is necessary to incorporate relevant strategies to promote mental well-being and target those who feel lonely. Thus, universities should provide teachers with resources to prevent mental health issues and needs-based counseling services.

There are several actions that university teachers can take to reduce or avoid the symptoms of anxiety and depression. For many teachers, primary health care measures such as engaging in regular exercise, obtaining enough quality sleep, eating a healthy diet, and avoiding alcohol can also help to reduce symptoms of anxiety and depression. For those university teachers with anxiety and depression symptoms, proper diagnosis and treatment are crucial.

This study is not without its limitations. First and foremost, the purposive sampling of universities and a focus on specific disciplines could affect the validity and generalizability of the findings. Additionally, shorter versions of tools used to measure anxiety, depression, and loneliness may not consistently measure constructs or cover all aspects. Participants may also underreport symptoms due to the fear of professional repercussions. The self-reported data may not accurately reflect mental health status, and the cross-sectional study design cannot establish causation. Future researchers need to conduct longitudinal studies to determine causality and address pre-existing anxiety and depression. Also, the current study had a limited sample size of university teachers. Future studies should include larger sample sizes and more diverse samples of university teachers.

## Figures and Tables

**Table 1:** Participants characteristics and their association with anxiety symptoms (*n* = 148).

Variable	Feelings of Anxiety (α = 0.76)		ꭓ^2^	*p* Value	Cramér’s V
	No Anxiety	Anxiety Symptom			
Overall	128 (89%)	16 (11%)			
Gender					
Women	22	3	0.03	0.866	0.01
Men	107	13			
Age ^1^					
Below 40 years	75	11	2.93	0.087	−0.15
40 and above	49	2			
Teaching experience					
<10 years of experience	69	12	2.67	0.102	−0.14
≥10 years of experience	60	4			
Academic rank					
Assistant professors	90	12	0.34	0.842	0.05
Associate professors	32	3			
Professors	6	1			
Instructional Type					
Blended	79	8	1.05	0.306	0.07
In-person	46	8			
University Location					
Centre or Capital	41	3	1.14	0.285	0.09
Regional State	88	13			
Loneliness					
Not having feelings of loneliness	124	7	44.76	0.000	0.56
Having feelings of loneliness	5	9			

Note: The n for each variable may not sum up to the total sample, where *n* = 148 due to random missing data. ꭓ^2^—chi-square. ^1^ Age was categorized into five groups based on the career stage theory [48].

**Table 2:** Participant characteristics and association with depressive symptoms (*n* = 148).

Variable	Feelings of Depression (α = 0.83)		ꭓ^2^	*p* Value	Cramér’s V
	No Depression	Depression Symptom			
Overall	128 (88.7%)	17 (12.3%)			
Gender					
Women	20	5	1.64	0.200	0.11
Men	108	13			
Age ^1^					
Below 40 years	75	12	3.44	0.064	−0.16
40 and above	49	2			
Teaching experience					
<10 years of experience	71	11	0.20	0.651	−0.04
≥10 years of experience	57	7			
Academic rank					
Assistant professors	89	14	0.63	0.730	0.07
Associate professors	32	3			
Professors	6	1			
Instructional Type					
Blended	81	7	2.54	0.111	0.13
In-person	45	9			
University Location					
Centre or Capital	41	3	1.77	0.183	0.11
Regional State	87	15			
Loneliness					
Not having feelings of loneliness	119	13	7.84	0.005	0.23
Having feelings of loneliness	9	5			

Note: The n for each variable may not sum up to the total sample, where *n* = 148 due to random missing data. ꭓ^2^—chi-square. ^1^ Age was categorized into five groups based on the career stage theory [48].

**Table 3:** Summary of binary regression predicting symptoms of anxiety and depression.

Variable	Anxiety Symptoms			Depression Symptoms		
	Odds Ratio	95% CI	*p*-Value	Odds Ratio	95% CI	*p*-Value
Loneliness	31.89	8.41, 120.83	0.000	5.09	1.48, 17.47	0.010

Note: *n* = 148. Anxiety symptom: coefficient = 3.46; depression symptom: coefficient: 1.63.

## Data Availability

The data that support the findings of this study are available from the primary author upon reasonable request.
